# Perinatal and Neonatal Outcomes of Patients Who Were Diagnosed with Neural Tube Defect in Midtrimester Fetal Ultrasound Scan and Refused Request for Termination of Pregnancy

**DOI:** 10.1155/2016/6382825

**Published:** 2016-11-23

**Authors:** Rauf Melekoglu, Sevil Eraslan, Ebru Celik, Yavuz Simsek

**Affiliations:** ^1^Department of Obstetrics and Gynecology, Faculty of Medicine, The University of Inonu, 44280 Malatya, Turkey; ^2^Private Clinic of Yavuz Simsek, 71200 Kırıkkale, Turkey

## Abstract

*Objectives*. In this study, we aimed to demonstrate the perinatal and neonatal outcomes of patients who were diagnosed with neural tube defect (NTD) in the midtrimester fetal ultrasound scan and refused the request for termination of pregnancy.* Material and Methods*. The records of 69 patients, for whom NTD was detected in the midtrimester fetal ultrasound scan and who preferred the continuation of the pregnancy after comprehensive counselling about the possible prognosis and treatment options during the period between January 2011 and February 2016, were reviewed retrospectively.* Results*. Of these patients, 66.7% were 25–35 years old; 95.7% were multiparous; and 1.4% had a history of a fetus having NTD in previous pregnancies. There were 7 (10.1%) neonatal deaths in these patients. Meningomyelocele closure procedure was the most performed surgery in the postnatal period (92%). Of these patients, 30.7% had paraplegia; 51.6% had neurogenic bladder; and 6.4% had infections due to surgery.* Conclusion*. The results of this study demonstrated perinatal and neonatal outcomes of fetuses with NTD who were not terminated by the preference of the family in midtrimester. The experience of our centre would be beneficial as a tool for nondirective counselling of these patients when considering the antenatal/postnatal care options and postnatal prognosis.

## 1. Introduction

Neural tube defects (NTD) are the most common malformations of central nervous system, and the incidence of NTD has been reported in about 9,65/10000. The incidence of NTD has been decreased over the years due to the termination of affected pregnancies and increased periconceptional folic acid use [1]. Maternal serum alpha-fetoprotein (AFP) measurement and ultrasound are effective screening methods for the diagnosis of NTD. Many factors can affect the results of AFP, so the routine midtrimester ultrasound screening is more efficient for the diagnosis of NTD [2]. Due to the dependence of prognosis to the level of the lesion, defining of lesion localization with ultrasonography (USG) and magnetic resonance imagining (MRI) is useful in predicting short and long-term outcomes [3]. Despite the advances in fetal and postnatal therapy, the option of elective pregnancy termination has still been considered vigorously with the family in prenatal counselling period. Although it was thought that NTD is a devastating disease associated with severe disability and reduced quality of life, it is a fact that many children and adults today with open spina bifida have a happy and productive life. The perception and comment about the possible fetal prognosis vary depending on every family's social dynamics and the cultural and economic environment [4]. The mission of obstetricians is to provide evidence-based information and options to the family by avoiding directive guidance.

In this study, we aimed to demonstrate the perinatal and neonatal outcomes of patients who were diagnosed with neural tube defects in the midtrimester fetal ultrasound scan and refused the request for termination of pregnancy.

## 2. Material and Methods

Inonu University Faculty of Medicine Ethics Committee consent was obtained before the study. The records of 69 patients were reviewed retrospectively for whom neural tube defect in the midtrimester fetal ultrasound scan were detected and preferred continuation of the pregnancy after comprehensive counselling about the possible prognosis and treatment options during the period between January 2011 and February 2016 at Inonu University School of Medicine Department of Obstetrics and Gynecology. Patients who met the following criteria were enrolled in this study:

1. Maternal age between 18 and 39
2. Singleton viable pregnancy
3. The detection of fetal neural tube defect between 14 weeks 24 weeks of gestation in obstetric ultrasonography
4. Patients who refused the option of pregnancy termination after comprehensive counselling

In the presence of the following situations the patients were excluded from the study:

1. Multiple pregnancies
2. Fetal death
3. Associated fatal congenital anomalies or chromosomal abnormalities

Antenatal care and delivery of patients included in this study were carried out in our clinic according to the standard protocols. Fetal MRI was not routinely performed in these patients due to its limited contribution to the diagnosis and prognosis in fetuses with neural tube defects. Postnatal MRI was preferred instead for providing more detailed information before surgery. All neonates with NTD were transferred to the neonatal intensive care unit (NICU), and after the cerebral and spinal magnetic resonance imaging, pediatric neurosurgery consultation was performed for the operation. Data were obtained from the medical records of patients and their neonates.

Descriptive characteristics were calculated for the variables of interest. Continuous and categorical variables were measured as median and mean with standard deviation. Quantitative data were summed up as number and percentile. Eligibility of data to the normal distribution was analyzed with Shapiro-Wilk test. Comparison of independent samples between the cesarean section and vaginal delivery groups was performed by *t*-test. Pearson's chi-square, Yate's corrected chi-square, and Fisher's exact tests were used for the analysis of categorical variables, where appropriate. Statistical analyses were performed using SPSS software (Statistical Package for the Social Sciences, Version 20, SPSS Inc., Chicago, IL).

## 3. Results

Data were collected from 69 patients who were diagnosed with neural tube defects in the midtrimester fetal ultrasound scan and preferred continuation of the pregnancy during the period between January 2011 and February 2016 at Inonu University School of Medicine Department of Obstetrics and Gynecology. Of these patients, 66.7% were between 25 and 35 years old; 95.7% were multiparous; 1.4% had a history of a fetus having NTD in previous pregnancies; 79.8% used periconceptional folic acid; and 18.8% had consanguinity with their spouse. Median gestational age at diagnosis was 20 (min 15–max 24) and 91.6% of cases admitted for the reference due to abnormal ultrasonography finding. Of these patients, 4.3% had cervical; 1.4% had thoracal; 13.0% had thoracolumbar; 34.8% had lumbar; 40.7% had lumbosacral; and 5.8% had sacral spina bifida (Table 1). The lemon sign was detected in 89.8% of these patients, the banana sign was detected in 85.5% of these patients, ventriculomegaly was detected in 92.7% of patients, and pes equinovarus deformity was detected in 21.7% of these patients in the midtrimester fetal ultrasound scan. Obstetric ultrasonography revealed associated anomalies in 3 (4.3%) of patients. One of these patients had cleft lip and palate; one had a perimembranous ventricular septal defect; and one had omphalocele.

Median gestational age at delivery was 38 (min 28–max 40) and 79.7% of these patients delivered by cesarean section. The presence of neural tube defects was the most common indication for cesarean delivery (Table 2).

Neonatal outcomes are demonstrated in Table 3. There were 7 (10.1%) neonatal death in these patients. Neonatal deaths were attributable to cardiac arrest (57.1%), prematurity (28.5%), and asphyxia (14.4%). The rate of neonatal morbidity was 36.2%, and the most observed neonatal morbidity was respiratory distress syndrome (88%). The median time of operation was one day (mean 1.67; range 1–21). Meningomyelocele closure procedure was the most performed surgery in the postnatal period (92%). The overall ventriculoperitoneal shunt requirement rate was detected as 33.8%. Of these patients who underwent a surgical procedure, 30.7% had paraplegia; 51.6% had a neurogenic bladder; and 6.4% had infections due to surgery (Table 3).

The neonatal outcomes of fetuses with NTD including neonatal mortality, neonatal morbidity, ventriculoperitoneal shunt requirement, lower extremity dysfunction, neurogenic bladder, and infections due to surgery were analyzed based on the level of the neural tube defect. It was determined that frequency of adverse neonatal outcomes was increased in accordance with the level of lesion. Neonatal outcomes of fetuses with NTD in midtrimester fetal ultrasound scan were summarized in Table 4 based on lesion level.

When the data was analyzed according to the delivery mode, it was demonstrated that cesarean delivery was not found to provide a superior benefit regarding neonatal mortality, neonatal morbidity, ventriculoperitoneal shunt requirement, lower extremity dysfunction, neurogenic bladder, and infections due to surgery. There was no statistically significant difference in adverse neonatal outcomes between the cesarean section and vaginal delivery group. Neonatal outcomes of fetuses with NTD in midtrimester fetal ultrasound scan were summarized in Table 5 based on delivery mode.

## 4. Discussion

The incidence of neonates with NTD has been decreased over the years due to the widespread use of folic acid in periconceptional period, the increment in prenatal diagnosis facilities, and preference of pregnancy termination. Despite advances in fetal and neonatal therapy options, most of the parents opt for the termination of pregnancy due to uncertainty in prenatal and neonatal prognosis. But the decision about the termination of pregnancy is affected by several factors, primarily sociocultural and religious situation. In this study, we demonstrated that neonatal mortality rate was 10.1% and found the overall ventriculoperitoneal shunt requirement rate was 33.8%, paraplegia rate was 30.7%, neurogenic bladder rate was 51.6%, and infection rate was 6.4% after the surgical procedure. And these adverse neonatal outcomes were found correlated with the lesion level. Bowman et al. reported the early adulthood survival rate as 75–80% and noted that it depends on the level of the lesion [5]. Cochrane et al. also categorised functional outcome of children by the level of spina bifida, for the purpose of correlation with prenatally diagnosed lesion level and prenatal counselling [6]. They found that while patterns of ambulation, urinary and bowel continence, and school performance vary according to the level of spinal lesion, the need for ventricular shunts, the incidence of other spinal malformations, and surgical interventions did not change with the level of the spinal lesion. Aygün et al. reported the experience of their centre on 100 newborns with neural tube defects [7]. They found hydrocephalus was associated in 67%, and of these, ventriculoperitoneal (VP) shunt was applied in 88% of babies. They observed neurological deficits (loss of strength, loss of sense, fecal incontinence, and neurogenic bladder) in 62% of infants, orthopaedic malformations in 54% of patients, and bladder dysfunction in 42% of patients.

Ultrasound has become the gold standard prenatal diagnostic tool for NTDs. In this study, we showed that intracranial findings of open spina bifida were present in most of the patients as the lemon sign was detected in 89.8%, the banana sign was detected in 85.5%, ventriculomegaly was detected in 92.7%, and pes equinovarus deformity was detected in 21.7% of these patients in midtrimester fetal ultrasound scan. Norem et al. reported the detection rates by ultrasound, ranging from 96 to 100% [2]. Nicolaides et al. reported the banana sign and lemon sign as the intracranial finding of open spina bifida in 1986 [8]. The banana sign is defined as the bending of the cerebellum into the posterior fossa and the lemon sign is described as the invaginating of the frontal bones as a result of shifting the intracranial contents from the foramen magnum. Van den Hof et al. reported 95% of fetuses with an open myelomeningocele having banana sign or absence of cerebellum on ultrasound regardless of gestational age compared with the lemon sign, which was present in 98% of fetuses 24 weeks or less but in only 13% of fetuses over 24 weeks [9]. They noted that despite nearly 100% of neonates born with an open myelomeningocele having evidence of hydrocephalus at the time of birth, only 70% had hydrocephalus during fetal life.

Associated anomalies other than those secondary to NTDs vary in different studies, according to their method of detecting additional abnormalities. In this study, we showed the rate of associated anomaly as 4.3%. Ekin et al. evaluated the frequency and types of associated anomalies with the results of ultrasonographic and postmortem examination and they reported 34.1% of NTD cases had associated morphological abnormalities [10]. They reported that skeletal anomalies were the most frequent (12.4%) and renal, cardiac, abdominal wall, and facial defects also have a higher frequency of the associated anomalies. Also, they found the prenatal detection rate of additional anomalies with ultrasonography was 73.1% compared with the autopsy findings. Other studies demonstrated the most frequent anomalies seen in all NTD cases were skeletal anomalies followed by renal, cardiac, abdominal wall, and facial defects [11–13].

In this study, we demonstrated that the mode of delivery in 79.7% of patients with NTD's was the cesarean section, and the presence of neural tube defects was the most common indication for cesarean delivery. Also, we observed that delivery mode was not associated with adverse neonatal outcomes. Inconsistently, some observational studies suggesting that cesarean delivery may provide a better outcome for babies with meningomyelocele (MMC) existed [14]. These studies suggested that cesarean delivery would be beneficial by keeping MMC sac intact, reducing bacterial contamination of the exposed neural tissue and also allow for a better preparation of the team (i.e., neonatologist and pediatric neurosurgeons). For these reasons, many centres adopted routine cesarean delivery for pregnancies complicated with NTD. The evidence supporting this recommendation is not strong. Merrill et al. detected no differences between either immediate or long-term outcome for the infant with isolated meningomyelocele when stratified by the mode of delivery [15]. In the present day, many professional organisations no longer consider fetal MMC as an absolute indication for cesarean delivery [16, 17].

Adzick et al. conducted a randomized controlled trial with 183 patients that opted for the continuation of pregnancy with the diagnosis of NTD for the evaluation of the benefit of in utero surgery. They found that the rates of adverse neonatal outcomes were similar between the in utero repair group and postnatal surgery group. Due to increased pregnancy complications related to the prenatal surgery including oligohydramnios, chorioamniotic separation, placental abruption, and spontaneous membrane rupture, the trial was stopped by the data and safety monitoring committee. Compared with our cohort, they experienced less neonatal mortality (2%) but more infection in the postnatal surgery group. The improvement in the neonatal mortality rate was probably associated with the inclusion criteria of their study that fetuses only myelomeningocele with the upper boundary located between T1 and S1 were enrolled in their study [18].

## 5. Conclusion

The results of this study demonstrated the perinatal and neonatal outcomes of fetuses with NTD who were not terminated by the preference of the family in midtrimester. Patients who were diagnosed with NTD in prenatal period should be counselled about the fetal and neonatal treatment options and referred to a centre that provides this specialised service. Comprehensive counselling of these patients about the perinatal and neonatal prognosis in a nondirective manner is crucial. In our country, although the termination of pregnancy is available as an option for fetuses with NTD, most women choose to continue their pregnancies regardless of lesion level, despite the possibility of significant disability and the potential need for long-term assisted care because of cultural and regional reasons. Thus, detailed information of these patients about the prognosis is of particular importance. The experience of our centre would be beneficial as a tool for nondirective counselling of these patients when considering the antenatal/postnatal care options and postnatal prognosis.

## Figures and Tables

**Table 1:** Baseline maternal and pregnancy characteristics of patients diagnosed NTD in midtrimester fetal ultrasound scan.

	*n* = 69
Age (year)^*∗*^	
Younger than 25	10 (14.5)
25–34.9	46 (66.7)
35 or older	13 (18.8)
Number of prior pregnancies 20 weeks of gestation or greater^*∗*^	
0 (nulliparous)	3 (4.3)
1	18 (26.1)
2	16 (23.2)
3 or more	32 (46.4)
History of fetus having NTD in previous pregnancies^*∗*^	1 (1.4)
Periconceptional folic acid use^*∗*^	55 (79.8)
Consanguinity between spouses^*∗*^	13 (18.8)
Gestational age at diagnosis^*∗∗*^	20 (15–24)
Reason for admission^*∗*^	
Routine obstetric visit	2 (2.8)
Elevation in maternal AFP MoM levels	4 (5.6)
Referred due to abnormal USG finding	63 (91.6)
The level of neural tube defect^*∗*^	
Cervical	3 (4.3)
Thoracal	1 (1.4)
Thoracolumbar	9 (13.0)
Lumbar	24 (34.8)
Lumbosacral	28 (40.7)
Sacral	4 (5.8)
Detection of lemon sign in USG^*∗*^	62 (89.8)
Detection of banana sign in USG^*∗*^	59 (85.5)
Detection of ventriculomegaly in USG^*∗*^	64 (92.7)
Detection of pes equinovarus deformity in USG^*∗*^	15 (21.7)
Associated anomaly (excluding clubfoot and Chiari II findings)^*∗*^	3 (4.3)

^**∗**^Data are given as *n* (%).

^*∗∗*^Data are given as median [interquartile range].

**Table 2:** Perinatal outcomes of patients diagnosed with NTD in midtrimester fetal ultrasound scan.

	*n* = 69
Gestational age at delivery (week)^*∗*^	**38 (28–40) **
Mode of delivery^*∗∗*^	
Vaginal	**14 (20.3)**
Cesarean	**55 (79.7)**
Indication for cesarean section^*∗∗*^	
Previous cesarean delivery	**24 (43.6)**
Neural tube defects	**27 (49.1)**
Fetal distress	**3 (5.5)**
Umbilical cord prolapse	**1 (1.8)**

^**∗**^Data are given as median [interquartile range].

^**∗∗**^Data are given as *n* (%).

**Table 3:** Neonatal outcomes of patients diagnosed with NTD in midtrimester fetal ultrasound scan.

	*n* = 69
Birth weight (gr)^*∗*^	**3100 (1160–4600) **
Birth height (cm)^*∗*^	**49 (32–51) **
(1) minute APGAR score^*∗*^	**6 (4–9) **
(5) minute APGAR score^*∗*^	**8 (6–10)**
Gender^*∗∗*^	
Male	33 (47.8)
Female	36 (52.2)
Neonatal morbidity^*∗∗*^	**25 (36.2) **
Respiratory Distress Syndrome	22 (88)
Persistent Pulmonary Hypertension	1 (4)
Meconium Aspiration Syndrome	1 (4)
Necrotising Enterocolitis	1 (4)
Neonatal mortality^*∗∗*^	**7 (10.1) **
Causes of mortality^*∗∗*^	
Cardiac arrest	4 (57.1)
Prematurity	2 (28.5)
Asphyxia	1 (14.4)
Timing of operation (day)^*∗*^	**1 (1–21)**
The form of surgery performed^*∗∗*^	
Meningomyelocele repair procedure	57 (92)
Ventriculoperitoneal shunt	2 (3.2)
Split thickness skin graft closure	1 (1.6)
Encephalocele closure at the occipital region	2 (3.2)
Ventriculoperitoneal shunt requirement^*∗∗*^	21 (33.8)
Lower extremity function^*∗∗*^	
Normal	43 (69.3)
Paraplegia	19 (30.7)
Neurogenic bladder^*∗∗*^	32 (51.6)
Infections due to surgery^*∗∗*^	4 (6.4)

^**∗**^Data are given as median [interquartile range].

^**∗∗**^Data are given as *n* (%).

**Table 4:** Neonatal outcomes of patients diagnosed with NTD in midtrimester fetal ultrasound scan based on lesion level.

	Cervical (*n* = 3)	Thoracal (*n* = 1)	Thoracolomber (*n* = 9)	Lumbar (*n* = 24)	Lumbosacral (*n* = 28)	Sacral (*n* = 4)
Neonatal mortality^**∗**^	3 (100)	1 (100)	2 (22.2)	0 (0)	1 (3.5)	0 (0)
Neonatal morbidity^**∗**^	2 (66.6)	1 (100)	6 (66.6)	9 (37.5)	6 (21.4)	1 (25)
Ventriculoperitoneal shunt requirement^**∗**^	3 (100)	1 (100)	4 (44.4)	7 (29.1)	6 (21.4)	0 (0)
Paraplegia in lower extremity^**∗**^	1 (33.3)	1 (100)	6 (66.6)	6 (25)	5 (17.8)	0
Neurogenic bladder^**∗**^	0	0	6 (66.6)	14 (58.3)	11 (39.2)	1 (25)
Infections due to surgery^**∗**^	0	0	2 (22.2)	0	2 (7.1)	0

^**∗**^Data are given as *n* (%).

**Table 5:** Neonatal outcomes of patients diagnosed with NTD in midtrimester fetal ultrasound scan based on delivery mode.

	Cesarean section (*n* = 55)	Vaginal delivery (*n* = 14)	*p*
Neonatal mortality^**∗**^	6 (10.9)	1 (7.1)	0.330
Neonatal morbidity^**∗**^	21 (38.1)	4 (28.5)	0.741
Ventriculoperitoneal shunt requirement^**∗**^	18 (32.7)	3 (21.4)	0.115
Paraplegia in lower extremity^**∗**^	14 (25.4)	5 (35.7)	0.091
Neurogenic bladder^**∗**^	26 (47.2)	6 (42.8)	0.389
Infections due to surgery^**∗**^	3 (5.4)	1 (7.1)	0.573

^**∗**^Data are given as *n* (%).
